# LIPOTOXICITY PLAYS A KEY ROLE IN THE DEVELOPMENT OF ANGIOGENESIS AND MICROCIRCULATORY MODULATION IN MASLD SPECTRUM

**DOI:** 10.1590/S0004-2803.24612025-053

**Published:** 2025-10-27

**Authors:** André Bubna HIRAYAMA, Isabela Bodaczny TALIBERTI, Claudia Pinto Marques Souza de OLIVEIRA, Venâncio Avancini Ferreira ALVES

**Affiliations:** 1 Universidade de São Paulo, Faculdade de Medicina, Departamento de Patologia, São Paulo, SP, Brasil.; 2 Centro de Imuno-histoquímica, Citopatologia e Anatomia Patológica (CICAP), São Paulo, SP, Brasil.; 3 Hospital Alemão Oswaldo Cruz, São Paulo, SP, Brasil.; 4 HCor - Hospital do Coração, São Paulo, SP, Brasil.; 5 Universidade de São Paulo, Faculdade de Medicina, Departamento de Gastroenterologia, São Paulo, SP, Brasil.

**Keywords:** Liver, Steatohepatitis, angiogenesis, Fígado, esteato-hepatite, angiogênese

## Abstract

Chronic liver diseases (CLDs), particularly metabolic-associated steatotic liver disease (MASLD), represent a significant global health burden, with potential progression to fibrosis, portal hypertension, and hepatocellular carcinoma (HCC). This review explores the role of vascular remodeling and angioarchitecture in MASLD pathogenesis, emphasizing the importance of microvascular changes, endothelial dysfunction, and angiogenesis in disease progression. Hepatic steatosis leads to hepatocyte enlargement and sinusoidal compression, disrupting microcirculation and oxygenation. Lipotoxicity, which is driven by excess fatty acids and oxidative stress, triggers inflammation and vascular permeability, fostering a proangiogenic environment. Capillarization of liver sinusoidal endothelial cells (LSECs) and activation of hepatic stellate cells (HSCs) contribute to fibrosis and neovascularization. Angiogenesis, especially via VEGF and other cytokines, is implicated in the transition from steatosis to steatohepatitis (MASH) and ultimately HCC, even in non-cirrhotic livers. Notably, portal hypertension may develop early in MASLD, independent of cirrhosis. Given the central role of vascular alterations in MASLD, future therapies may target endothelial and stromal cell interactions. Further research is needed to delineate disease-specific mechanisms and their implications for fibrosis and carcinogenesis.

## HIGHLIGHTS

• MASLD involves significant vascular changes such as sinusoidal capillarization and angiogenesis.

• Lipid accumulation and hypoxia in hepatocytes trigger inflammatory and proangiogenic pathways, promoting liver injury and microvascular dysfunction early in disease development.

• Lipotoxicity and vascular alterations play a crucial role in disease progression and portal hypertension, representing promising targets for therapeutic strategies and further research.

## RESUMO

. As doenças hepáticas crônicas (DHCs), especialmente a doença hepática gordurosa associada ao metabolismo (MASLD), representam um ônus significativo para a saúde global, com potencial progressão para fibrose, hipertensão portal e carcinoma hepatocelular (CHC). Esta revisão explora o papel da remodelação vascular e da angioarquiteturana na patogênese da MASLD, enfatizando a importância das alterações microvasculares, da disfunção endotelial e da angiogênese na progressão da doença. A esteatose hepática leva ao aumento dos hepatócitos e à compressão sinusoidal, interrompendo a microcirculação e a oxigenação. A lipotoxicidade, impulsionada pelo excesso de ácidos graxos e estresse oxidativo, desencadeia inflamação e permeabilidade vascular, favorecendo um ambiente pró-angiogênico. A capilarização das células endoteliais sinusoidais do fígado (LSECs) e a ativação das células estreladas hepáticas (HSCs) contribuem para a fibrose e a neovascularização. A angiogênese, especialmente via VEGF e outras citocinas, está implicada na transição da esteatose para a esteato-hepatite (MASH) e, finalmente, para o CHC, mesmo em fígados não cirróticos. Notavelmente, a hipertensão portal pode se desenvolver precocemente na MASLD, independentemente da cirrose. Dado o papel central das alterações vasculares na MASLD, futuras terapias podem ter como alvo as interações entre células endoteliais e estromais. Mais pesquisas são necessárias para delinear os mecanismos específicos da doença e suas implicações para a fibrose e a carcinogênese.

## INTRODUCTION

Chronic liver diseases (CLD) are widespread and deadly worldwide, pose a significant burden on public health, and are mostly associated with cirrhosis and its clinical complications, such as portal hypertension, hepatic failure, and progression to hepatocellular carcinoma, requiring major clinical and surgical intervention, even as liver transplantation. The liver, a highly vascular organ, exhibits vascular changes, including vascular remodeling, sinusoidal capillarization and intrahepatic shunts, in these diseases. These alterations lead to increased hepatic resistance and decreased hepatocyte perfusion^1^.

Angiogenesis is the process of the formation of new vessels from preexisting tissue and occurs not only under physiological conditions, such as during wound healing or pregnancy but also under pathological conditions, such as neoplastic processes^2^. After a proangiogenic signal, such as VEGF, angiopoietin-2 or FGF, endothelial cells become activated and proliferate, with an increase in endovascular permeability, changes in the extracellular matrix and the migration of endothelial cells, which lead to the formation of new vessels^3^.

Since CLD is not a uniform entity, alterations in angioarchitecture along the progression of each disease follows some broad, generic mechanisms as well as several pathways more specifically related to each original disease, such as viral hepatitis, vascular diseases and metabolic-associated steatotic liver disease (MASLD). Nowadays, following increasing rates of obesity and diabetes worldwide, MASLD is one of the most prevalent CLDs globally, particularly affecting obese and type 2 diabetes patients^2^ ^,^ ^4^ ^-^ ^6^. While histological finding of steatosis without further inflammation appears to most usually follow a favorable clinical course, MASH syndrome is recognized as a potentially progressive disease that may cause end-stage liver disease and hepatocellular carcinoma^7^ ^-^ ^9^.

Additionally, in CLD, hepatocellular carcinoma (HCC), a well-vascularized tumor, develops, as some patients with MASLD develop HCC without advanced fibrosis, indicating that alterations in the microvasculature may contribute to carcinogenesis^10^ ^-^ ^12^.

This review aims to evaluate the role of angioarchitecture in the etiopathology of MASLD, focusing on the pathogenesis, potential therapeutic options and, finally, the development of HCC.

## Fat accumulation and changes in microvascular circulation

The liver is a core element in lipid metabolism and plays an important role in the uptake, esterification, oxidation and export of fatty acids^13^. Additionally, these processes are regulated by multiple factors, such as hormones, nuclear receptors and transcription factors^14^.

The uptake of circulating fatty acids is highly dependent on FATP (fatty acid transporter protein), CD36 and caveolin in the hepatocyte membrane^14^ ^,^ ^15^. Dietary triglycerides are sent to peripheral tissues and the liver through a process mediated by LDL receptors. The lipids do not circulate freely in the cytosol and are transported and stored by FATP, with isoform 1 being predominant in the liver^14^ ^,^ ^16^.

Additionally, hepatocytes also convert carbohydrates to lipids by *de novo* lipogenesis, and these processes are upregulated by insulin^13^. The accumulation of lipid droplets in the cytoplasm causes the enlargement of hepatocytes, which may exacerbate injury by depriving centrilobular regions of oxygen and nutrients^17^.

Seifalian et al. first evaluated microcirculatory alterations using laser Doppler flowmetry and showed that macroscopically steatotic livers had diminished microcirculation compared with that of normal livers^18^. Additionally, researchers used a rabbit model to assess how much steatosis compromises sinusoidal circulation, focusing on early graft dysfunction in transplants. They reported that hepatic parenchymal microcirculation flow was significantly reduced in steatotic livers, mainly in livers with higher grades of steatosis^19^.

In a mouse model of a high-fat methionine - and choline-deficient diet and in vivo microscopy, McCuskey et al. showed that dramatically enlarged lipid-laden hepatocytes started compressing the lumen of sinusoids, mainly in the centrilobular regions, giving sinusoids irregular contours with low flow rates, which was also related to an initial increase in phagocytic activity, with a late reduction in Kupffer cell activity^17^.

It is also important to emphasize the role of liver sinusoidal endothelial cells (LSECs) in the process of injury: LSECs regulates the interaction between the circulation and the hepatocytes, having a distinct phenotype (without basement membrane and with open fenestrae), being highly permeable cells. Liver injury induce alterations in LSECs, who acquire a capillarized phenotype, losing its fenestrate and gaining a basal membrane, which reduces the oxygenation and promotes a hypoxic microenvironment. These capillary-like endothelial cells secrete pro-inflammatory, profibrotic, prothrombotic, and proangiogenic mediators, creating a cellular network that contributes to increased portal pressure and fibrosis progression. Endothelial dysfunction is an early phenomenon in MASLD pathogenesis^20^ ^-^ ^22^.

**FIGURE 1:**
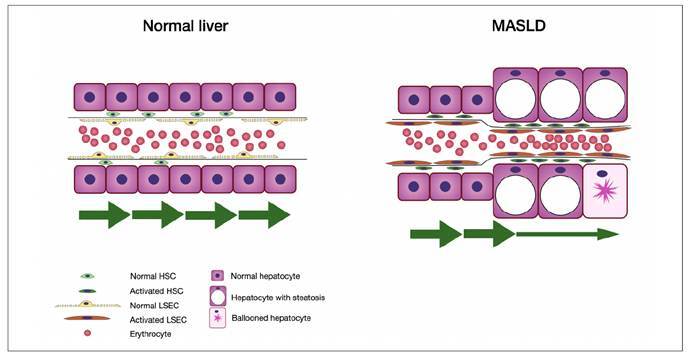
In normal liver microcirculation, sinusoids are delicate with fenestrated liver sinusoidal endothelial cells (LSEC) and quiescent hepatic stellate cells (HSC) (left). In metabolic dysfunction-associated steatotic liver disease (MASLD), mechanical and oxidative stress from steatotic and ballooned hepatocytes alters the morphology of LSEC, causing them to lose their fenestrae and adopt a capillarized phenotype. This also activates HSCs and reduces sinusoidal blood flow (right).

As sinusoidal capillarization and extracellular matrix accumulation occur, sinusoidal stiffness and shear stress increase, leading to greater mechanical activation, cell injury, and progressive fibrosis (Figure 1). Experimental models have shown that portal hypertension increases chemokine secretion by neutrophils, such as CXCL1, promoting leukocyte recruitment and the formation of neutrophil extracellular traps, which favor hepatic microvascular thrombosis, further increasing portal pressure and contributing to fibrosis^23^ ^,^ ^24^.

## Lipotoxicity

Lipotoxicity occurs when the capacity of the liver to store and export fatty acids such as triglycerides is overwhelmed by the flux from the periphery, which is correlated with disease severity^25^. A high content of saturated fatty acids also promotes endoplasmic reticulum stress and oxidative stress^25^ ^-^ ^28^. Angiogenesis is a factor-dependent growth process, and inflammation and hypoxia are key factors involved in its induction^29^.

Additionally, uncontrolled oxidative stress causes direct damage to lipids and lipid peroxidation, which activates hepatic stellate cells (promoting angiogenesis), recruits Kupffer cells and increases cytokine secretion^30^.

Through the accumulation of lipids in the cytoplasm of hepatocytes, lipotoxicity triggers cytokine production, inflammatory cell recruitment and increased vascular permeability, which can initiate vascular changes in the liver. Hypoxia and angiogenesis are closely related, with the first promoting the second.

The presence of steatosis increases the sensitivity of hepatocytes to hypoxia, and this effect is reversed by defatting^31^. Hypoxia exacerbates lipotoxicity, which is a link between steatosis and steatohepatitis and contributes to crosstalk with Kupffer cells^32^ ^,^ ^33^.

## The progression to steatohepatitis and fibrosis

Recently, the role of angiogenesis in the progression of MASLD has become more relevant: although not restricted to MASLD, angiogenesis is expected to play a key role in progression, as it is centered in the perivenular zone, which is more susceptible to ischemia and hypoxia^2^. Kitade et al. (2006) showed in a mouse model that leptin-mediated neovascularization plays a role in VEGF and oxidative stress in the progression of steatohepatitis, fibrogenesis and HCC^34^.

The serum levels of IL-6 and VEGF were higher in patients with a histological diagnosis of MASH than in patients presenting only steatosis or a normal liver^35^. Researchers compared the levels of inflammatory and angiogenic cytokines in biopsies between patients with MASH and patients with simple steatosis and normal liver tissue, revealing increased expression of TNAα, IL6, VEGF and sVEGFR1 in a population with steatosis and MASH compared to healthy controls and greater expression of TNAα in MASH patients than in patients with steatosis^36^.

In two murine models, Coulon et al. (2013) showed an increase in VEGF in the early stages of MASH, with an increase in the progression from steatosis to MASH^37^. Additionally, it is well known that the development of fibrosis parallels the increase in vascularization, as both stimulate each other^38^ ^-^ ^41^.

In a mouse model, impaired sinusoidal blood flow was related to the deposition of collagen and extracellular matrix in the space of Disse during the release of ROS, which could explain the “capillarization” of sinusoids as an early event in steatohepatitis progression^17^.

After hepatocyte injury, hepatic stellate cells, which undergo myofibroblastic differentiation, exhibit a proangiogenic phenotype and secrete angiopoietin-1^42^ ^-^ ^44^. Additionally, as HSCs proliferate, they cover sinusoids and stimulate angiogenesis and fibrogenesis^45^.

## Portal hypertension

Portal hypertension has been observed in liver disease associated with MASLD even in the absence of cirrhosis or significant fibrosis. The hepatic venous pressure gradient (HVPG) is the main parameter used to assess portal pressure, with normal values ranging from 1 to 5 mmHg. Portal hypertension is defined as HVPG >5 mmHg, with values >10 mmHg indicating clinically significant portal hypertension, and values >12 mmHg associated with higher risk of complications and decompensations. In MASLD patients with cirrhosis, HVPG may underestimate actual portal pressure, with even less accuracy in the absence of cirrhosis, requiring more reliable methods. Subclinical portal hypertension (HVPG 6.0-9.5 mmHg) is often found in MASLD patients, even without cirrhosis or significant fibrosis, frequently associated with steatosis or mild to moderate fibrosis. Although its clinical impact is not fully understood, evidence suggests this elevated portal pressure may contribute to liver disease progression^22^ ^,^ ^46^.

## The beginning of carcinogenesis

Angiogenesis not only supports the growth of solid tumors but is also an early step in carcinogenesis^47^. Hypervascularization is a hallmark of HCC due to its high demands for oxygen and nutrients, with fast-growing tumors having a proangiogenic signature^48^ ^,^ ^49^. In the MASLD model, blockade of angiopoietin-2 halted HCC progression in a mouse model^50^.

## Perspectives

The vascular changes in MASLD play a central role in the beginning and progression of disease, being a potential target for future therapeutic interventions. Also, this review highlights the need for new studies in this field, with a focus in endothelial cells and myofibroblasts, as these cells probably are not mere supporting cells, but maybe have a key role in the vasculogenesis. Special attention is required to possible differences in mechanisms more directly related to each etiological condition as well as to the recently defined histological subtypes of HCC.
